# Yearling laryngeal function grades II.2 and below are not associated with reduced performance

**DOI:** 10.1111/evj.14452

**Published:** 2025-01-21

**Authors:** Josephine L. Hardwick, Benjamin J. Ahern, Kylie L. Crawford, Kate J. Allen, Brian H. Anderson, Kim J. Rose, Samantha H. Franklin

**Affiliations:** ^1^ School of Animal and Veterinary Sciences University of Adelaide Adelaide South Australia Australia; ^2^ School of Veterinary Medicine Murdoch University Perth Western Australia Australia; ^3^ School of Veterinary Science University of Queensland Brisbane Queensland Australia; ^4^ Mater Research Institute University of Queensland Brisbane Queensland Australia; ^5^ Bristol Veterinary School Bristol University Bristol UK; ^6^ Ballarat Veterinary Practice Lake Wendouree Victoria Australia; ^7^ Equine Sales Services Perth Western Australia Australia

**Keywords:** horse, larynx, racehorse, RLN, sale, upper airway

## Abstract

**Background:**

The relationship between Thoroughbred yearling laryngeal function (YLF) grade and race performance is unclear.

**Objectives:**

To determine the effect of YLF on future race performance.

**Study design:**

Retrospective cohort study.

**Methods:**

Post‐sale endoscopic recordings were reviewed from Australian yearling sales in 2018–2019. Race performance was evaluated for career and as 2‐, 3‐ and ≥4‐year‐olds. Multivariable generalised linear modelling examined the association between YLF and performance, with risk estimates presented as coefficients (95% CI).

**Results:**

The YLF in 5175 examinations was graded I in 29.8% (*n* = 1542); II.1 in 49.0% (*n* = 2537), II.2 in 16.5% (*n* = 855), III.1 in 3.9% (*n* = 200), III.2 in 0.8% (*n* = 39) and III.3 in <0.04% (*n* = 2). Additional endoscopic abnormalities included ventroaxial luxation of the corniculate process (VLAC, *n* = 77, 1.5%); arytenoid mucosal lesions (*n* = 392, 7.6%) and intermittent dorsal displacement of the soft palate (iDDSP, *n* = 1264, 24.4%). Median (IQR) career earnings were: grade I $45 095 ($15 565, $113 220); grade II.1 $45 315 ($15 915, $107 490), grade II.2 $38 610 ($14 326, $95 218), grade III.1 $32 765 ($8565, $86 030) and grade III.2 $35 810 ($3700, $65 770). There was no difference in career earnings for YLF grades II.2 and III.1, compared with referent grade I/II.1, whereas grade III.2 earned less overall (−$46 015 (95% CI: −$89 994, −$2036), *p* = 0.04). Earnings in ≥4‐year‐olds were less for grades III.1 (−$35 076 (−$56 129, −$14 024), *p* = 0.001) and III.2 (−$53 219, (−$76 062, −$30 375) *p* < 0.001).

**Main limitations:**

Lack of follow‐up data due to retrospective nature of study. Exclusion of unraced horses and those with no prize money from analysis.

**Conclusions:**

Ninety‐five percent of the yearling population had grades I, II.1 or II.2 YLF and minimal difference in race performance was identified between them. Horses with grade III.1 YLF performed similarly to grades I/II.1 in their early careers but had reduced race performance at ≥4‐year‐old.

## 1 INTRODUCTION

Videoendoscopic assessment of a yearling's upper respiratory tract is considered an important part of the pre‐sale examination process and the yearling laryngeal function (YLF) grade assigned can have significant economic implications, including an effect on sales price and clearance rate.^1^, ^2^, ^3^, ^4^ In Australia, YLF is graded on a 5‐point scale, but the published description of a grade 3 used in the Conditions of Sale by Australian sales companies^5^, ^6^ differs from that in a later publication of the same grading system.^7^ While ostensibly minor, this difference has contributed to confusion and variability among veterinarians at yearling sales.^1^ Elsewhere a more granular 7‐point (Havemeyer) grading system is used.^8^ This allows horses that achieve full symmetrical arytenoid cartilage abduction, but display prolonged asymmetry of the rima glottidis (grade 3 out of 5)^7^ to be separated into two categories: those that can maintain full arytenoid cartilage abduction (grade II.2), and those that cannot maintain full arytenoid cartilage abduction (grade III.1).^8^

In Australia, there is significant industry concern surrounding the future race performance of yearlings identified with grade 3/5 YLF, due to conflicting data on the effect on future race performance.^3^, ^4^, ^9^ A North American study identified horses with grade II.2 YLF had reduced earnings as a 4‐year‐old,^3^ whereas a similar Australian study found the earnings of horses with grades II.2 and III.1 YLF were not significantly different from grades I and II.1 YLF.^4^ Neither of the aforementioned studies adjusted for confounders in their analysis.^10^, ^11^ A recent study investigating YLF in horses that underwent a prosthetic laryngoplasty, identified almost half of the horses that required a prosthetic laryngoplasty had grades I and II.1 YLF. However, multivariable analysis revealed the risk of surgery did increase from YLF grade II.2 and upwards. No race performance data was analysed in that study.^12^

The aim of this study was to assess the association between YLF grade and race performance indices. We hypothesised that horses with ≥grade II.2 YLF would have reduced future performance compared with grades I/II.1 YLF.

## 2 MATERIALS AND METHODS

### 2.1 Study design

This was a retrospective cohort study. Post‐sale videoendoscopic examinations acquired at Australian Thoroughbred yearling sales were obtained from three equine clinics. Sales data including sex, year of birth and sales price were located from online sales records (www.inglis.com.au; www.magicmillions.com.au). Race names were obtained from online records. Race performance history was sourced through The Rating Bureau (www.trb.com.au).

### 2.2 Inclusion criteria

To be included in the study, horses had to have an available post‐sale videoendoscopic examination obtained at a 2018 or 2019 yearling sale. Cases were excluded if videos were considered to be non‐diagnostic for the following reasons: there was inadequate visualisation of the larynx to evaluate arytenoid abduction following at least three swallows, poor video quality and if no consensus in YLF grade was achieved between three observers. Cases were also excluded if any additional non‐recurrent laryngeal neuropathy (RLN) related endoscopic abnormalities were identified that would not meet the conditions of sale, including arytenoid chondritis or chondropathy; right‐sided laryngeal dysfunction; rostral displacement of the palatopharyngeal arch, permanent dorsal displacement of the soft palate and sub‐epiglottic cysts.^6^ Additionally, an Australian race record was required to be included in the performance analysis.

### 2.3 Grading of videoendoscopic examinations

The videos were graded by two independent observers out of a team of six observers. Each observer was not privy to other observer grades (i.e., blinded). If there was disagreement in the YLF grade assigned by the initial two observers, a third observer was used to provide a consensus YLF grade for further analysis. In cases where consensus in the YLF grade was not provided by the third observer, these horses were excluded from the study. All observers were experienced in grading YLF, and consisted of 3 specialist equine surgeons, 2 specialists in equine sports medicine and 1 prominent sales veterinarian.

For each video the following observations were recorded: YLF grade using the 7‐point grading system^8^; intermittent dorsal displacement of the soft palate (iDDSP, yes/no); mucosal lesions of the arytenoid cartilages (left/right/bilateral/no)^13^ and ventro‐medial luxation of the apex of the corniculate process of the arytenoid cartilage (VLAC, left under right/right under left/bilateral/no).^14^, ^15^ Concurrent endoscopic abnormalities (iDDSP, VLAC and mucosal lesions) were considered present in the analysis if at least one observer had identified them in the video.

Due to previously identified disagreement between veterinarians when grading laryngeal function, a more objective method of grading YLF was used, known as the diagnostic decision tree (DDT).^12^, ^16^ The DDT has been described elsewhere, but briefly it is based on the framework of the Havemeyer grading system and consists of a series of dichotomous (yes/no) decisions, that lead the observer down a pathway towards the most appropriate laryngeal function grade. The DDT contains several key objective definitions, most notably the decision between horses with prolonged arytenoid cartilage asymmetry (grades II.2 and III.1). A set of 40 endoscopic videos recorded at 25 frames/s and previously assigned a unanimous grade of II.2 or III.1 by three experienced observers, was used to determine the cut‐off between grade II.2 and III.1.^16^ For horses with prolonged arytenoid cartilage asymmetry, *maintained* full symmetrical arytenoid abduction (grade II.2) was defined as horses that could achieve and maintain full abduction for a mean of ≥0.2 s (or ≥5 frames, when recorded at 25 frames/s). Whereas full symmetrical abduction that is achieved but *not maintained* (grade III.1) was defined as being able to maintain full abduction for a mean of <0.2 s. The diagnostic decision tree is displayed in Figure S1.

### 2.4 Data analyses

#### 2.4.1 Sample size calculations

There are no appropriate studies investigating the relationship between YLF grade and these performance indices to base sample size calculations thus, sample size calculations were not performed. A previous study using the Havemeyer grading system, grouped the performance of YLF grade III.1 together with grades III.2 and III.3 and no spread of the dispersion of data points was provided.^3^ Power was deemed adequate after assessing 95% confidence interval widths. Narrow confidence intervals suggest the study has enough precision to detect an effect, if it exists.

#### 2.4.2 Exposure

The exposure, YLF grade, was measured using the 7‐point grading system.^8^ For the multivariable analyses, grades I and II.1 were combined, as they are both considered to represent normal YLF.^3^, ^4^

#### 2.4.3 Outcomes

Racing performance indices evaluated included prizemoney, prizemoney per start, peak rating, number of starts, number of wins and number of places. Each of these outcomes was analysed for the total career, as a 2‐year‐old, 3‐year‐old and ≥4‐year‐old.

#### 2.4.4 Covariates

Covariates included in multivariable models were clinically relevant confounders of the association between laryngeal function grade and performance indices, because recent methodological papers have demonstrated the superiority of models using this approach.^10^, ^11^ Confounders were determined by specialist consensus, and included sex, yearling sale, sales price and concurrent endoscopic abnormalities.

#### 2.4.5 Univariable and multivariable analyses

The distribution of continuous variables was assessed using histograms. Population characteristics were presented, stratified by laryngeal function grade. The different yearling sales were anonymised and assigned a numerical sale code. Descriptive data were described as median (IQR) when data were not normally distributed. For each laryngeal function grade, median earnings were presented as a 2‐year‐old, 3‐year‐old, ≥4‐year‐old and the total career. Univariable generalised linear models (inverse Gaussian family with canonical link) were used to assess the unadjusted association between laryngeal function grade and performance indices, as the outcome data did not meet the assumptions of normality required for standard linear regression. The link function allows the model to function like a linear regression model. Subsequently, multivariable models adjusting for sex, yearling sale and concurrent endoscopic abnormalities were performed. Due to a high correlation between sale and sale price, we adjusted for sale, rather than both. For all models, unraced horses and those that earned no prizemoney were excluded, as modelling a large number of zero counts necessitates statistical techniques such as negative binomial regression analysis and these models often fit poorly with extreme outliers. Thus, it was considered more robust to exclude these from the analyses. For all models, robust standard errors (Huber–White Sandwich estimator) were used to account for unknown clustering that could lead to a lack of independence among observations, such as sale conditions and variations in endoscopic examination techniques. Model specification was assessed using deviance residuals and histograms of deviance residuals.^17^ Risk estimates were presented as coefficients and 95% confidence intervals (95% CI), whereby a coefficient represents the increase or decrease in each performance index for that laryngeal function grade compared with the mean of the referent category of grades I and II.1. A coefficient >1 represents that many units increase in performance for that grade compared with the mean of grades I and II.1, whereas a coefficient <1 represents a decrease. For example, a coefficient of 110 represents $110 more earnings for that grade compared with grades I and II.1, whereas a coefficient of −110 represents $110 less earnings for that grade compared with grades I and II.1. Significance was set at *α* = 0.05 for all statistical tests. The reporting of this study conforms to the STROBE (Strengthening the Reporting of Observational Studies in Epidemiology) statement.^18^ Statistical analyses were performed using Stata 18 (Statacorp LLC).

#### 2.4.6 Inter‐rater agreement

The frequencies of assigned laryngeal grades and the percentages of agreement between observers were calculated.

## 3 RESULTS

### 3.1 Recruitment data

**FIGURE 1:**
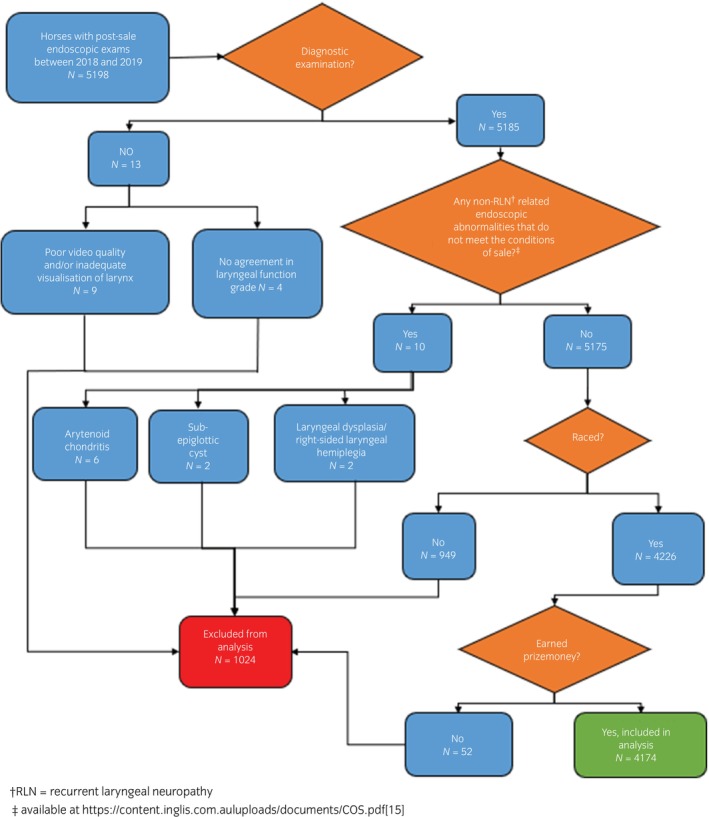
Flow chart detailing horses that were excluded from the performance analysis.

A total of 5198 post‐sale endoscopic examinations, from 8 different yearling sales, were graded by the team of observers, with 23 excluded due to non‐diagnostic recordings or other non‐RLN related findings that did not meet the conditions of sale. Of the remaining 5175 horses, 4174 horses were included in the final performance analysis. A flow chart of case exclusions is displayed in Figure 1.

### 3.2 Population characteristics

**FIGURE 2:**
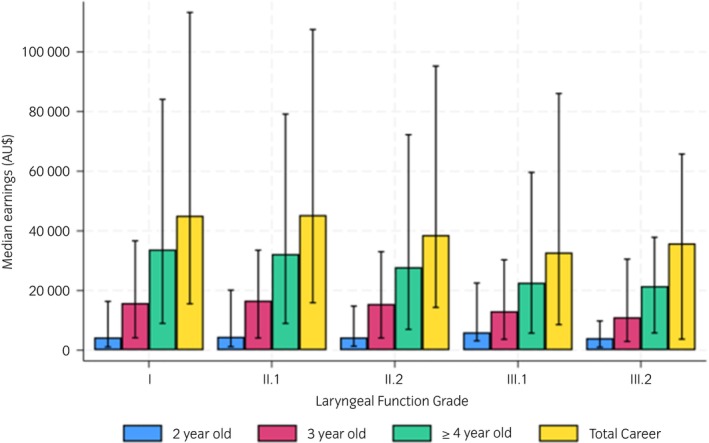
Median earnings in AU$ (IQR) for each laryngeal function grade as a 2‐year‐old, 3‐year‐old, ≥4‐year‐old and total career.^8^

For the 5175 horses included in the study, demographic data, concurrent endoscopic abnormalities, and race performance history stratified by YLF grade are displayed in Table 1. The YLF was graded I in 29.8% (*n* = 1542); II.1 in 49.0% (*n* = 2537), II.2 in 16.5% (*n* = 855), III.1 in 3.9% (*n* = 200), III.2 in 0.8% (*n* = 39), III.3 in 0.04% (*n* = 2) and no horses were grade IV. The median earnings (IQR) for each laryngeal function grade as a 2‐year‐old, 3‐year‐old, ≥4‐year‐old and for total career are displayed in Figure 2. The data were heavily skewed due to a select group of elite horses with exceptional performance and a modest number of horses with very poor earnings.

**TABLE 1:** Population characteristics stratified by yearling laryngeal function grade.

Yearling laryngeal function grade^a^	I	II.1	II.2	III.1	III.2	III.3	Total
	*N* = 1542	*N* = 2537	*N* = 855	*N* = 200	*N* = 39	*N* = 2	*N* = 5175
Sale information							
Sale code							
1	164 (10.6%)	362 (14.3%)	87 (10.2%)	28 (14.0%)	2 (5.1%)	0 (0.0%)	643 (12.4%)
2	198 (12.8%)	257 (10.1%)	148 (17.3%)	28 (14.0%)	6 (15.4%)	0 (0.0%)	637 (12.3%)
3	276 (17.9%)	300 (11.8%)	72 (8.4%)	22 (11.0%)	3 (7.7%)	1 (50.0%)	674 (13.0%)
4	452 (29.3%)	793 (31.3%)	288 (33.7%)	63 (31.5%)	7 (17.9%)	0 (0.0%)	1603 (31.0%)
5	139 (9.0%)	278 (11.0%)	81 (9.5%)	12 (6.0%)	6 (15.4%)	1 (50.0%)	517 (10.0%)
6	117 (7.6%)	245 (9.7%)	89 (10.4%)	24 (12.0%)	10 (25.6%)	0 (0.0%)	485 (9.4%)
7	139 (9.0%)	214 (8.4%)	55 (6.4%)	15 (7.5%)	5 (12.8%)	0 (0.0%)	428 (8.3%)
8	57 (3.7%)	88 (3.5%)	35 (4.1%)	8 (4.0%)	0 (0.0%)	0 (0.0%)	188 (3.6%)
Year of sale							
2018	650 (42.2%)	979 (38.6%)	336 (39.3%)	72 (36.0%)	17 (43.6%)	1 (50.0%)	2055 (39.7%)
2019	892 (57.8%)	1558 (61.4%)	519 (60.7%)	128 (64.0%)	22 (56.4%)	1 (50.0%)	3120 (60.3%)
Sex							
Female	732 (47.5%)	1123 (44.3%)	380 (44.4%)	86 (43.0%)	13 (33.3%)	0 (0.0%)	2334 (45.1%)
Male	810 (52.5%)	1414 (55.7%)	475 (55.6%)	114 (57.0%)	26 (66.7%)	2 (100.0%)	2841 (54.9%)
Sale purchase price	60 000 (26 000, 160 000)	70 000 (30 000, 160 000)	60 000 (26 000, 150 000)	75 000 (30 000, 160 000)	40 000 (20 000, 100 000)	12 000 (12 000, 12 000)	65 000 (28 000, 160 000)
Concurrent abnormalities							
VLAC^b^	11 (0.7%)	30 (1.2%)	26 (3.0%)	8 (4.0%)	2 (5.1%)	0 (0.0%)	77 (1.5%)
Mucosal lesions^c^	104 (6.7%)	193 (7.6%)	69 (8.1%)	24 (12.0%)	2 (5.1%)	0 (0.0%)	392 (7.6%)
iDDSP^d^	269 (17.4%)	635 (25.0%)	266 (31.1%)	78 (39.0%)	14 (35.9%)	2 (100.0%)	1264 (24.4%)
Racing and performance history							
Un‐named	181 (11.7%)	296 (11.7%)	121 (14.2%)	28 (14.0%)	8 (20.5%)	2 (100.0%)	636 (12.3%)
Unraced	273 (17.7%)	458 (18.1%)	167 (19.5%)	41 (20.5%)	8 (20.5%)	2 (100.0%)	949 (18.3%)
Starts in career	17 (8, 27)	17 (8, 28)	16 (7, 25)	13 (7, 22)	11 (4, 20)	^e^	16 (8, 27)
Starts as a 2 year old	0 (0, 2)	0 (0, 2)	0 (0, 2)	0 (0, 2)	0 (0, 0)	^e^	0 (0, 2)
Starts as a 3 year old	5 (3, 8)	5 (2, 8)	5 (2, 7)	4 (2, 7)	3 (2, 6)	^e^	5 (2, 8)
Starts as ≥4 years old	9 (2, 20)	10 (2, 20)	9 (2, 18)	8 (1, 15)	6 (1, 14)	^e^	9 (2, 19)
Did not start as a 2 year old	688 (54.2%)	1099 (52.9%)	384 (55.8%)	95 (59.7%)	24 (77.4%)	^e^	2290 (54.2%)
Did not start as a 3 year old	124 (9.8%)	230 (11.1%)	88 (12.8%)	15 (9.4%)	4 (12.9%)	^e^	461 (10.9%)
Did not start as a ≥4 year old	243 (19.1%)	406 (19.5%)	131 (19.0%)	37 (23.3%)	7 (22.6%)	^e^	824 (19.5%)
Prizemoney in career	45 095 (15 565, 113 220)	45 315 (15 915, 107 490)	38 610 (14 326, 95 218)	32 765 (8565, 86 030)	35 810 (3700, 65 770)	^e^	43 682 (14 713, 105 625)
Prizemoney as a 2 year old	4300 (1100, 16 350)	4500 (1175, 20 122)	4300 (1350, 14 798)	5985 (3115, 22 500)	4036 (1020, 9800)	^e^	4500 (1200, 17 458)
Prizemoney as a 3 year old	15 800 (4149, 36 650)	16 600 (4092, 33 505)	15 500 (4100, 33 010)	13 075 (3650, 30 290)	11 030 (2930, 30 500)	^e^	15 880 (4055, 34 260)
Prizemoney as ≥4 years old	33 745 (8980, 84 070)	32 260 (8960, 79 139)	27 810 (7000, 72 235)	22 638 (5710, 59 600)	21 488 (5762, 37 852)	^e^	31 862 (8270, 78 445)
No prizemoney	17 (1.3%)	27 (1.3%)	4 (0.6%)	3 (1.9%)	1 (3.2%)	^e^	52 (1.2%)
No prizemoney as a 2 year old	26 (4.5%)	45 (4.6%)	9 (3.0%)	1 (1.6%)	0 (0.0%)	^e^	81 (4.2%)
No prizemoney as a 3 year old	23 (1.8%)	44 (2.2%)	12 (1.8%)	4 (2.5%)	1 (3.2%)	^e^	84 (2.0%)
No prizemoney as a ≥4 year old	26 (2.5%)	33 (2.0%)	14 (2.5%)	3 (2.5%)	1 (4.2%)	^e^	77 (2.3%)
Prizemoney/start in career	2718 (1286, 5108)	2667 (1276, 4876)	2424 (1266, 4764)	2515 (1080, 4525)	2055 (853, 3534)	^e^	2638 (1259, 4912)
Prizemoney/start as a 2 year old	2000 (712, 5870)	1945 (724, 6120)	1902 (794, 5685)	2664 (1125, 7984)	2018 (510, 2450)	^e^	2000 (736, 6000)
Prizemoney/start as a 3 year old	2625 (1017, 5594)	2770 (1021, 5359)	2522 (1027, 5200)	2796 (860, 4925)	2758 (1156, 3360)	^e^	2704 (1012, 5329)
Prizemoney/start as ≥4 years old	2420 (1017, 4720)	2328 (1004, 4305)	2139 (912, 4035)	2034 (836, 3971)	2219 (926, 3938)	^e^	2306 (992, 4390)
Wins in career	2 (0, 4)	2 (1, 4)	1 (0, 3)	1 (0, 3)	1 (0, 3)	^e^	2 (0, 4)
Wins as a 2 year old	0 (0, 0)	0 (0, 0)	0 (0, 0)	0 (0, 0)	0 (0, 0)	^e^	0 (0, 0)
Wins as a 3 year old	0 (0, 1)	0 (0, 1)	0 (0, 1)	0 (0, 1)	0 (0, 1)	^e^	0 (0, 1)
Wins as ≥4 years old	1 (0, 2)	1 (0, 2)	0 (0, 2)	0 (0, 1)	0 (0, 1)	^e^	1 (0, 2)
Wins in career = 0	320 (25.2%)	517 (24.9%)	190 (27.6%)	51 (32.1%)	12 (38.7%)	^e^	1090 (25.8%)
Wins as a 2 year old = 0	1130 (89.0%)	1809 (87.0%)	618 (89.8%)	140 (88.1%)	30 (96.8%)	^e^	3727 (88.2%)
Wins as a 3 year old = 0	666 (52.5%)	1088 (52.3%)	379 (55.1%)	85 (53.5%)	21 (67.7%)	^e^	2239 (53.0%)
Wins as ≥4 years old = 0	591 (46.6%)	977 (47.0%)	352 (51.2%)	92 (57.9%)	18 (58.1%)	^e^	2030 (48.0%)
Places in career	5 (2, 11)	5 (2, 10)	5 (2, 9)	4 (1, 8)	3 (1, 8)	^e^	5 (2, 10)
Places as a 2 year old	0 (0, 0)	0 (0, 1)	0 (0, 0)	0 (0, 0)	0 (0, 0)	^e^	0 (0, 0)
Places as a 3 year old	2 (0, 3)	2 (0, 3)	1 (0, 3)	1 (0, 2)	1 (0, 3)	^e^	2 (0, 3)
Places as ≥4 years old	3 (0, 7)	3 (0, 7)	2 (0, 6)	1 (0, 5)	1 (0, 3)	^e^	2 (0, 7)
Places in career = 0	160 (12.6%)	297 (14.3%)	102 (14.8%)	33 (20.8%)	6 (19.4%)	^e^	598 (14.2%)
Places as a 2 year old = 0	968 (76.3%)	1547 (74.4%)	532 (77.3%)	121 (76.1%)	27 (87.1%)	^e^	3195 (75.6%)
Places as a 3 year old = 0	382 (30.1%)	668 (32.1%)	226 (32.8%)	54 (34.0%)	10 (32.3%)	^e^	1340 (31.7%)
Places as ≥4 years old = 0	413 (32.5%)	709 (34.1%)	244 (35.5%)	69 (43.4%)	11 (35.5%)	^e^	1446 (34.2%)
Peak rating in career	84 (77, 90)	84 (77, 89)	83 (76, 89)	83 (76, 88)	81 (72, 87)	^e^	84 (77, 89)
Peak rating as a 2 year old	79 (71, 86)	80 (72, 86)	80 (72, 87)	82 (74, 86)	78 (62, 87)	^e^	80 (72, 86)
Peak rating as a 3 year old	82 (75, 88)	82 (74, 88)	81 (73, 87)	82 (75, 87)	80 (73, 84)	^e^	82 (74, 88)
Peak rating as ≥4 years old	82 (74, 89)	82 (74, 88)	80 (74, 87)	81 (73, 86)	78 (72, 85)	^e^	82 (74, 88)

*Note*: Data are presented as median (IQR) for continuous measures and *n* (column %) for categorical measures.

^a^ Laryngeal function grade (Dixon et al. 2003).^8^

^b^ VLAC = ventromedial luxation of the apex of the corniculate process of the arytenoid (Barakzai et al. 2007).^14^

^c^ Mucosal lesions (Kelly et al. 2003).^13^

^d^ iDDSP = dorsal displacement of the soft palate.

^e^ Not applicable as un‐raced.

### 3.3 Generalised linear models

Models were built for all performance indices. The univariable results are displayed in Table S1. The multivariable results are displayed in Table 2. Due to consistent trends in the data suggesting biological plausibility of the findings and the fact that our findings are supported by other literature,^3^ no correction of statistical significance was performed. Sex and yearling sale were strongly associated with performance indices, as well as YLF. Additionally, they confounded the effect of YLF for the majority of performance outcomes.

**TABLE 2:** Multivariable generalised linear models of the effect of yearling laryngeal function grade^8^ on performance indices, adjusted for sex, yearling sale and concurrent endoscopic abnormalities.

Laryngeal function grade	Referent mean	Coefficient^a^	(95% CI)		*p*‐value
Total career earnings AU$					
I and II.1	110 923	Referent	Referent	Referent	Referent
II.2	—	−1862	−30 523	26 798	0.9
III.1	—	−23 438	−60 985	14 108	0.2
III.2	—	−46 015	−89 994	−2036	0.04
Two year old earnings AU$					
I and II.1	27 793	Referent	Referent	Referent	Referent
II.2	—	−4295	−16 641	8052	0.5
III.1	—	−5070	−25 293	15 152	0.6
III.2	—	18 896	−117 023	154 816	0.8
Three year old earnings AU$					
I and II.1	36 512	Referent	Referent	Referent	Referent
II.2	—	5208	−7074	17 491	0.4
III.1	—	7357	−17 508	32 223	0.6
III.2	—	−14 495	−31 152	2162	0.09
≥4 year old earnings AU$					
I and II.1	82 734	Referent	Referent	Referent	Referent
II.2	—	−3504	−27 615	20 608	0.8
III.1	—	−35 076	−56 129	−14 024	0.001
III.2	—	−53 219	−76 062	−30 375	<0.001
Earnings AU$ per start career					
I and II.1	5409	Referent	Referent	Referent	Referent
II.2	—	347	−1259	1953	0.7
III.1	—	−406	−2788	1976	0.7
III.2	—	−951	−4554	2652	0.6
Earnings AU$ per start 2 years old					
I and II.1	7253	Referent	Referent	Referent	Referent
II.2	—	−69	−2372	2234	>0.9
III.1	—	−426	−4586	3735	0.8
III.2	—	2250	−13 063	17 562	0.8
Earnings AU$ per start 3 years old					
I and II.1	5245	Referent	Referent	Referent	Referent
II.2	—	2539	−889	5967	0.2
III.1	—	2056	−3426	7538	0.5
III.2	—	−846	−6092	4399	0.8
Earnings AU$ per start ≥ 4 years old					
I and II.1	4923	Referent	Referent	Referent	Referent
II.2	—	−396	−1620	828	0.5
III.1	—	−1075	−2815	665	0.2
III.2	—	−1793	−4313	727	0.2
Peak rating career					
I and II.1	82.0	Referent	Referent	Referent	Referent
II.2	—	−0.7	−1.7	0.2	0.1
III.1	—	−0.9	−2.7	0.9	0.3
III.2	—	−4.0	−7.8	−0.2	0.04
Peak rating 2 years old					
I and II.1	77.5	Referent	Referent	Referent	Referent
II.2	—	0.4	−1.3	2.0	0.7
III.1	—	0.8	−2.6	4.2	0.7
III.2	—	−0.8	−10.3	8.7	0.9
Peak rating 3 years old					
I and II.1	80.1	Referent	Referent	Referent	Referent
II.2	—	−0.8	−1.8	0.2	0.1
III.1	—	−0.2	−2.2	1.7	0.8
III.2	—	−2.4	−6.6	1.8	0.3
Peak rating ≥4 years old					
I and II.1	80.2	Referent	Referent	Referent	Referent
II.2	—	−0.4	−1.5	0.7	0.5
III.1	—	−0.9	−3.1	1.2	0.4
III.2	—	−2.8	−7.4	1.7	0.2
Number of starts career					
I and II.1	18.9	Referent	Referent	Referent	Referent
II.2	—	−1.4	−2.4	−0.5	0.003
III.1	—	−3.1	−4.7	−1.6	<0.001
III.2	—	−6.1	−8.5	−3.6	<0.001
Number of starts 2 years old					
I and II.1	2.7	Referent	Referent	Referent	Referent
II.2	—	−0.3	−0.5	−0.1	0.003
III.1	—	0.1	−0.41	0.5	0.8
III.2	—	−0.04	−1.4	1.3	>0.9
Number of starts 3 years old					
I and II.1	6.0	Referent	Referent	Referent	Referent
II.2	—	−0.4	−0.7	−0.1	0.006
III.1	—	−0.6	−1.1	−0.1	0.01
III.2	—	−1.0	−2.0	0.05	0.06
Number of starts ≥4 years old					
I and II.1	15.2	Referent	Referent	Referent	Referent
II.2	—	−1.0	−1.9	−0.2	0.02
III.1	—	−3.0	−4.3	−1.7	<0.001
III.2	—	−5.6	−7.6	−3.7	<0.001
Number of wins career					
I and II.1	3.1	Referent	Referent	Referent	Referent
II.2	—	−0.2	−0.4	0.01	0.07
III.1	—	−0.4	−0.7	−0.01	0.04
III.2	—	−0.8	−1.4	−0.2	0.006
Number of wins 2 years old					
I and II.1	1.4	Referent	Referent	Referent	Referent
II.2	—	−0.01	−0.2	0.2	0.9
III.1	—	−0.2	−0.5	0.03	0.09
III.2	—	0.5	−1.9	3.0	0.7
Number of wins 3 years old					
I and II.1	1.6	Referent	Referent	Referent	Referent
II.2	—	−0.1	−0.2	0.1	0.4
III.1	—	−0.1	−0.3	0.2	0.6
III.2	—	0.1	−0.52	0.73	0.7
Number of wins ≥4 years old					
I and II.1	2.6	Referent	Referent	Referent	Referent
II.2	—	−0.1	−0.3	0.2	0.6
III.1	—	−0.3	−0.6	0.1	0.1
III.2	—	−0.7	−1.3	−0.1	0.02
Number of places career					
I and II.1	7.7	Referent	Referent	Referent	Referent
II.2	—	−0.4	−0.8	0.1	0.1
III.1	—	−1.0	−1.8	−0.2	0.01
III.2	—	−2.1	−3.4	−0.8	<0.001
Number of places 2 years old					
I and II.1	1.9	Referent	Referent	Referent	Referent
II.2	—	−0.1	−0.3	0.1	0.4
III.1	—	−0.1	−0.5	0.2	0.5
III.2	—	0.6	−1.2	2.3	0.5
Number of places 3 years old					
I and II.1	3.0	Referent	Referent	Referent	Referent
II.2	—	−0.1	−0.3	0.03	0.1
III.1	—	−0.3	−0.6	0.03	0.08
III.2	—	−0.4	−1.1	0.2	0.2
Number of places ≥4 years old					
I and II.1	6.3	Referent	Referent	Referent	Referent
II.2	—	−0.3	−0.7	0.1	0.2
III.1	—	−1.1	−1.7	−0.4	0.002
III.2	—	−3.0	−3.6	−2.3	<0.001

*Note*: Risk estimates were presented as coefficients (95% CI).

Abbreviation: AU$, Australian dollars.

^a^ A coefficient >1 represents that many units increase in performance for that grade compared with the mean of grades I and II.1, whereas a coefficient <1 represents a decrease.

There was no difference in total career earnings for YLF grades II.2 and III.1, compared with referent grade I/II.1, whereas grade III.2 earned less overall (−$46 015 (95% CI: −$89 994, −$2036), *p* = 0.04). Earnings for ≥4‐year‐old were less for grades III.1 (−$35 076 (−$56 129, −$14 024), *p* = 0.001) and III.2 (−$53 219, (−$76 062, −$30 375) *p* < 0.001), compared with the referent grade I/II.1. The number of career starts was less for YLF grade II.2 (−1.4 starts (−2.4, −0.5), *p* = 0.003), grade III.1 (−3.1 starts (−4.7, −1.6), p < 0.001) and grade III.2 (−6.0 starts (−8.5, −3.6), p < 0.001), compared with the referent grade I/II.1. There were less career wins for grade III.1 (−0.4 wins (−0.7, −0.01), *p* = 0.04) and grade III.2 (−0.8 wins (−1.4, −0.2), *p* = 0.006). There were fewer career places for grade III.1 (−1.0 (−1.8, −0.2), *p* = 0.01) and grade III.2 (−2.1 (−3.4, −0.8), *p* < 0.001).

### 3.4 Percentage of agreement

Excluding cases with poor video quality (*n* = 9) and those with non‐RLN related abnormalities (*n* = 10), a consensus YLF grade was assigned in 5175 cases (99.9%) and consensus in YLF grade was not reached in 4 cases (<0.1%). A consensus YLF grade was classified as the grade assigned by both observers 1 and 2, or in cases of disagreement, the consensus grade was the one assigned by a third observer that agreed with either observer 1 or observer 2. Out of the 5175 cases with a consensus grade, identical YLF grades were assigned to the same endoscopic video by observers 1 and 2 in 3441 horses (66.5%), and they differed by 1 grade in 1662 horses (32.1%), by 2 grades in 70 horses (1.4%) and by 3 grades in 2 horses (<0.1%). The most frequent disagreements were between grades I and II.1 in 1108 horses (63.9% of the differences) and grades II.1 and II.2 (410 horses, 23.6%). There were a further 124 (7.2%) disagreements between grades II.2 and III.1, and smaller proportions whereby there were differences across two or more grades.

## 4 DISCUSSION

The results of this study showed that there was no difference in future race performance between 95% of the horses examined (YLF ≤grades II.2). The only exception was horses with grade II.2 YLF started a mean of 1.4 races less over their career than grades I and II.1; however, given there was no difference in earnings, number of wins and number of starts, this was considered to be of minimal clinical relevance. Our findings echo those of Ahern et al.,^4^ but they contrast those of Garrett et al.,^3^ who found horses with grade II.2 YLF earned significantly less as a 4‐year‐old, compared with grades I and II.1. A New Zealand study using the 5‐point scale, found no difference in performance parameters between yearlings with grade 3 out of 5 YLF (grades II.2 and III.1 combined), compared with less than grade 3 (≤grade II.1).^9^ The more granular 7‐point grading system separates grade 3 (out of 5) YLF into two distinct categories and our results support its use when evaluating YLF.

Horses with grade III.1 YLF (4% of the population) performed similarly to grades I and II.1 across most performance indices as a 2‐ and 3‐year‐old, including earnings, number of wins and number of places. However, they earned significantly less in their 4‐year‐old plus careers. This could be explained by the progressive nature and typical age of onset of clinical RLN, however, this cannot be substantiated, due to the lack of follow‐up data. Recurrent laryngeal neuropathy is a degenerative condition and the median age for onset of clinical signs in Thoroughbreds is 3–6 years.^12^, ^19^ Progressive loss of large myelinated alpha nerve fibres in the recurrent laryngeal nerve, leads to arytenoid cartilage collapse during exercise, resulting in exercise intolerance and poor performance.^20^, ^21^, ^22^

A previous study in the United States, using the Havemeyer grading system, found a significant difference in performance between horses with grade III and grade I YLF, however, that study grouped all grade III sub‐grades together due to low numbers.^3^ In contrast, a previous Australian study found no difference in performance between horses with grade III.1 and grades I/II.1 YLF, however, this study was likely underpowered and did not account for confounders.^4^ A recent study identified horses with grade III.1 YLF had a significantly increased risk of requiring a prosthetic laryngoplasty, which suggests these horses are indeed further along the disease pathway than lower laryngeal function grades.^12^ We are not aware if any horses in our study underwent a laryngoplasty.

The disparity in race performance of horses with grade II.2 and III.1 YLF between our study and others may be due to the use of different statistical methods. If data are not adjusted for confounders, it can lead to an overestimate or underestimate of the true association between the exposure (YLF grade) and the outcome (racing performance), or potentially even change the observed effect.^10^, ^11^ We adjusted for sex, because it is causally associated with both YLF grade and race performance, as RLN affects males more commonly than females.^23^ Gelded males have also been shown to have more race starts, higher career earnings and longer career durations than females.^23^, ^24^ Co‐linearity was identified between yearling sale and sales price, therefore we also adjusted the model for sale as premier sales attract horses with more desirable genetics and conformational traits, plus higher sales prices are positively associated with increased performance.^25^

Another explanation for differing associations between YLF grades II.2 and III.1 and future performance is the use of the DDT to grade YLF, rather than the original wording of the Havemeyer grading system.^8^, ^16^ Although the proportions of horses with each YLF grade in our population were similar to other sale populations, the DDT may be more consistent at correctly categorising laryngeal function.^3^, ^4^, ^26^ Use of the DDT resulted in a high level of agreement and reliability between two observers grading YLF in horses that underwent a prosthetic laryngoplasty compared with controls.^12^ Interpretation of the Havemeyer system has been shown to be subjective, particularly around the definition of whether or not full arytenoid cartilage abduction is maintained.^26^, ^27^ Whereas, the DDT provides an objective definition for both grade II.2 (abduction maintained) and grade III.1 (abduction not maintained).^16^

Less than 1% of the sales population had ≥grade III.2 YLF, however, the true prevalence of higher laryngeal function grades in yearlings is likely underrepresented. Pre‐sale endoscopic examinations, both on farm and at the sales complex, are common practice and yearlings identified with higher YLF grades are frequently withdrawn from sale, or do not meet their reserve price (‘pass‐in’), meaning no post‐sale endoscopic examination would be available.^1^ The reduction in multiple performance indices for yearlings that did not achieve full arytenoid cartilage abduction (≥grades III.2), supports the findings of others.^3^, ^4^ However, given the small number of yearlings with grade III.2 YLF that raced in the study and the large confidence intervals, results for this grade should be interpreted with caution. Excluding unraced horses and those that earned no prizemoney from the performance analysis, whilst statistically robust, may introduce bias and minimise the true impact of higher YLF grades on racing performance. A future prospective study with longitudinal follow‐up on these underperforming horses would be ideal, however this would require substantial time and financial resources.

A meta‐analysis of 12 studies found resting endoscopy to be a sensitive and highly specific at predicting exercising function in adult horses presenting almost exclusively with poor performance or respiratory noise during exercise.^28^ However, caution is required when extrapolating this data to an asymptomatic yearling Thoroughbred population, as clinical disease is unlikely to develop until after the commencement of race training and RLN is a progressive disease.^12^, ^29^ In a Thoroughbred population, the median age at time of prosthetic laryngoplasty surgery was 3 years of age and almost 50% of cases had YLF grades I or II.1 YLF, indicating deterioration in laryngeal function must have occurred to necessitate surgery.^12^ Very few studies have investigated the relationship between resting and exercising function in yearlings, likely because a sufficient level of training and maturity is required before undergoing an exercising endoscopic examination. In a UK study of 57 yearlings that all passed the conditions of sale and could achieve full arytenoid cartilage abduction at rest (≤grade III.1), 5% had abnormal exercising laryngeal function despite only being exercised at the canter.^30^ In another study of young Thoroughbreds during serial exercising endoscopic examinations, the severity of arytenoid cartilage collapse either progressed or remained unchanged in all cases.^31^ The lack of improvement in arytenoid cartilage collapse fits with RLN being a progressive condition. The findings of these two studies, coupled with a reported incidence of progression of laryngeal dysfunction in 5%–15% of cases, indicate that it is likely more than 5% of yearlings that meet the conditions of sale will develop arytenoid cartilage collapse at exercise.^29^, ^32^

Grades I and II.1 YLF were combined in our analysis, because no difference between these two grades has been shown in terms of race performance,^3^, ^4^ or the risk of requiring a prosthetic laryngoplasty.^12^ Additionally, disagreement between observers was most frequent between grades I and II.1. If observers cannot reliably differentiate between grades I and II.1, analysing the performance of these horses separately may be clinically irresponsible, particularly given industry concerns that some purchasers only want to buy horses with grade I YLF.^1^

Our results and those from another study, support monitoring yearlings identified with higher YLF grades for progressive loss of laryngeal function following sale.^12^ In clinical cases of RLN that can maintain partial arytenoid cartilage abduction during exercise, alternative surgical procedures to a prosthetic laryngoplasty may be beneficial and have fewer complications. These include removal of the vocal fold and/or laryngeal ventricle, or newer laryngeal reinnervation techniques.^33^, ^34^ The authors are unaware of any published data on surgical procedures in subclinical cases of RLN, however, there are anecdotal reports. Further research is required to identify which subclinical cases of RLN will progress into clinical disease and which will remain asymptomatic, to avoid unnecessary, and potentially detrimental, iatrogenic damage to the laryngeal musculature.

Concurrent endoscopic abnormalities had little effect on the relationship between YLF grade and race performance. The presence of VLAC in resting and exercising endoscopic examinations has been reported elsewhere, and it has been detected concurrently with arytenoid cartilage collapse, or other forms of dynamic upper airway collapse, during exercise.^14^, ^15^, ^35^ To the authors' knowledge, the prevalence of VLAC in a yearling population has not been previously reported. The effect of VLAC on future performance was a concern raised by sales veterinarians during a focus group on yearling endoscopy, but the significance of this abnormality is currently unknown and warrants further investigation.^1^

Multiple studies have identified a need to improve agreement between veterinarians when grading laryngeal function.^1^, ^26^, ^27^ Despite the 7‐point system being a well‐defined grading system, there is still subjectivity involved in the interpretation, particularly when determining if full symmetrical abduction is maintained. Grades I, II.1 and II.2 caused 87.5% of the disagreements and yet there was minimal difference in race performance between them, therefore disagreements between grade II.2 and below should be considered clinically less important than between grades II.2 and above. Two studies showed the use of a dichotomous classification (≤ grade II.2 or ≥grade III.1) improved interobserver agreement compared with an ordinal scale,^26^, ^27^ therefore it may be prudent for sales veterinarians to focus on whether full arytenoid cartilage abduction is maintained, given this was the main determinant of risk in our population.

### 4.1 Limitations

Due to the retrospective nature of this study, we were unable to account for horses that developed clinical RLN, underwent surgery and then had altered post‐operative performance. Regardless, this limitation has the advantage of making our findings more robust, as it shifts them toward the null. Another limitation is that many factors affect race performance, including musculoskeletal disease, lower respiratory disease, genetics and training factors, however, we could only adjust the model to account for known factors. We acknowledge the inherent limitations of excluding unraced horses and those that earned no prizemoney, but this reflects a real‐world situation. A future prospective cohort study to allow longitudinal follow‐up on these underperforming horses would be ideal, but this would likely be prohibitively long and costly.

## 5 CONCLUSIONS

The use of an objective definition of the term maintenance of abduction allowed us to identify that a small subset of yearlings (grade III.1) were at an increased risk of reduced future performance in their later race careers. However, these horses were still able to perform similarly to their ‘normal’ peers during their 2‐ and 3‐year‐old careers. Yearling laryngeal function grades II.2 and below were not associated with reduced future race performance. Although varying degrees of laryngeal asymmetry and asynchrony are common in yearlings, their presence is inconsequential to future race performance, providing that a yearling can achieve and maintain full arytenoid cartilage abduction (grade II.2). Accurate differentiation of grade II.2 YLF from grade III.1 YLF is an important diagnostic step for the sales veterinarian and using a reliable and objective method for this differentiation is indicated. The findings of this study are most applicable to Thoroughbred yearlings sold through Australian sales and may not be generalisable to other populations.

## FUNDING INFORMATION

This research was funded by AgriFutures Australia.

## CONFLICT OF INTEREST STATEMENT

The authors have declared no conflicting interests.

## AUTHOR CONTRIBUTIONS

**Josephine L. Hardwick:** Conceptualization; investigation; funding acquisition; writing – original draft; methodology; validation; visualization; formal analysis; project administration; data curation. **Benjamin J. Ahern:** Conceptualization; investigation; funding acquisition; methodology; validation; visualization; writing – review and editing; supervision; resources. **Kylie L. Crawford:** Methodology; validation; writing – review and editing; software; formal analysis; supervision; visualization. **Kate J. Allen:** Methodology; supervision; writing – review and editing; validation; investigation. **Brian H. Anderson:** Investigation; validation; writing – review and editing; supervision; methodology. **Kim J. Rose:** Investigation; resources; writing – review and editing; validation. **Samantha H. Franklin:** Investigation; conceptualization; funding acquisition; methodology; writing – review and editing; supervision; validation; visualization; project administration; resources.

## DATA INTEGRITY STATEMENT

Josephine L. Hardwick had full access to all the data in the study and takes responsibility for the integrity of the data and the accuracy of data analysis.

## ETHICAL ANIMAL RESEARCH

The University of Adelaide's animal ethics committee confirmed that ethical approval was not required due to it being a retrospective study.

## INFORMED CONSENT

Owners/agents were aware that medical records may be used for research in general.

### PEER REVIEW

The peer review history for this article is available at https://www.webofscience.com/api/gateway/wos/peer-review/10.1111/evj.14452.

## Supporting information


**Figure S1.** The diagnostic decision tree.

**Table S1.** Univariable generalised linear models of the effect of yearling laryngeal function grade on performance indices.

## Data Availability

The data that support the findings of this study are openly available in Figshare at https://figshare.com; reference number 28030772; doi:10.6084/m9.figshare.28030772.
