# Supplementary figures and images for: Yearling laryngeal function grades II.2 and below are not associated with reduced performance
Source: Equine Vet J. 2025 Jan 21;57(4):953–66. doi: 10.1111/evj.14452 (PMC12135752; doi:10.1111/evj.14452)

**Figure S1:** The diagnostic decision tree.

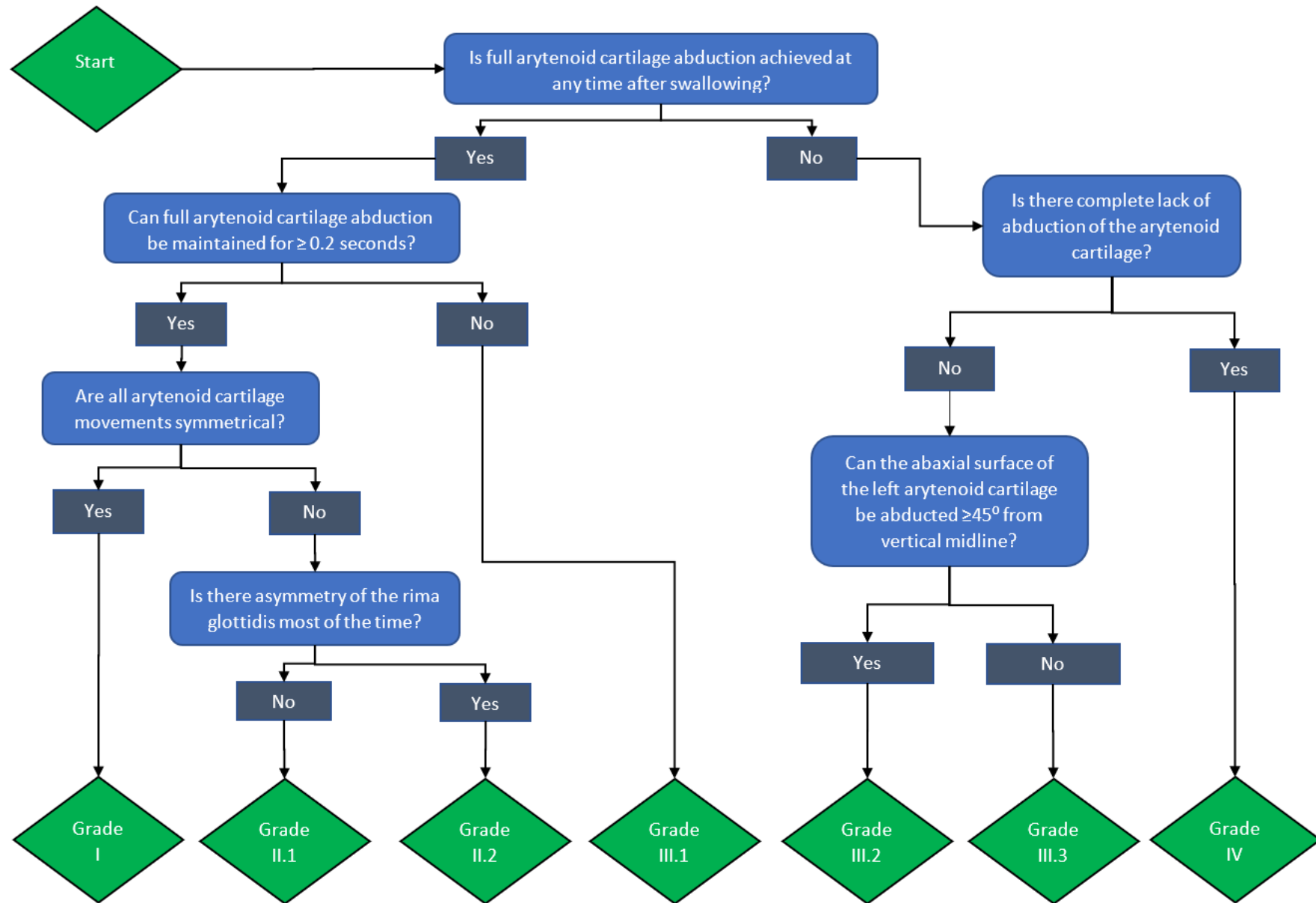

Supplement: Supplementary file 1 — Figure S1. The diagnostic decision tree. [file EVJ-57-953-s002.pdf]
