# Supplementary material for: Yearling laryngeal function grades II.2 and below are not associated with reduced performance
Source: Equine Vet J. 2025 Jan 21;57(4):953–66. doi: 10.1111/evj.14452 (PMC12135752; doi:10.1111/evj.14452)
Supplement: Supplementary file 2 — Table S1. Univariable generalised linear models of the effect of yearling laryngeal function grade on performance indices. [file EVJ-57-953-s001.pdf]

Table S1. Univariable generalised linear models of the effect of yearling laryngeal function grade [6] on performance indices. Risk estimates were presented as coefficients (95% CI).

Laryngeal Function Grade	Coefficient†	(95% CI)		p-value
**Total Career Earnings**				
I and II.1	Referent	Referent	Referent	Referent
II.2	-4,271	-30,727	22,186	0.8
III.1	-19,023	-60,554	22,508	0.4
III.2	-64,458	-99,137	-29,780	<0.001
**2yo Earnings**				
I and II.1	Referent	Referent	Referent	Referent
II.2	-365	-14,098	13,367	>0.9
III.1	-3,389	-26,798	20,021	0.8
III.2	94	-83,482	83,669	1.00
**3yo Earnings**				
I and II.1	Referent	Referent	Referent	Referent
II.2	7,512	-5,263	20,287	0.3
III.1	11,647	-17,219	40,513	0.4
III.2	-16,059	-34,444	2,326	0.09
**≥ 4yo Earnings**				
I and II.1	Referent	Referent	Referent	Referent
II.2	-11,418	-32,642	9,806	0.3
III.1	-35,245	-59,096	-11,394	0.004
III.2	-52,093	-79,442	-24,745	<0.001
**Earnings Per Start Career**				
I and II.1	Referent	Referent	Referent	Referent
II.2	841	-802	2,484	0.3
III.1	491	-2,526	3,508	0.8
III.2	-2,112	-4,991	766	0.2
**Earnings Per Start 2yo**				
I and II.1	Referent	Referent	Referent	Referent
II.2	146	-2,365	2,658	0.9
III.1	-494	-4,960	3,973	0.8
III.2	-1,926	-11125	7,274	0.7
**Earnings Per Start 3yo**				
I and II.1	Referent	Referent	Referent	Referent
II.2	3,432	-10	6,874	0.05
III.1	2,653	-3,374	8,680	0.4
III.2	-1,197	-6,272	3,878	0.6
**Earnings Per Start ≥ 4yo**				
I and II.1	Referent	Referent	Referent	Referent
II.2	-330	-1,567	907	0.6
III.1	-1,425	-3,089	239	0.09
III.2	-2,246	-4,698	205	0.07
**Peak Rating Career**				
I and II.1	Referent	Referent	Referent	Referent
II.2	-0.7	-1.7	0.2	0.1
III.1	-0.9	-2.7	1.0	0.4
III.2	-4.2	-8.0	-0.4	0.03
**Peak Rating 2yo**				
I and II.1	Referent	Referent	Referent	Referent
II.2	0.4	-1.3	2.0	0.7
III.1	1.3	-2.1	4.6	0.5
III.2	-1.4	-10.9	8.2	0.8
**Peak Rating 3yo**				
I and II.1	Referent	Referent	Referent	Referent
II.2	-0.8	-1.8	0.3	0.1
III.1	-0.2	-2.1	1.8	0.9

III.2	-2.7	-6.9	1.6	0.2
**Peak Rating ≥ 4yo**				
I and II.1	Referent	Referent	Referent	Referent
II.2	-0.4	-1.4	0.7	0.5
III.1	-0.9	-3.0	1.3	0.4
III.2	-2.7	-7.3	1.8	0.2
**Number of Starts Career**				
I and II.1	Referent	Referent	Referent	Referent
II.2	-1.6	-2.6	-0.6	0.002
III.1	-3.6	-5.2	-2.0	<0.001
III.2	-5.6	-8.4	-2.8	<0.001
**Number of Starts 2yo**				
I and II.1	Referent	Referent	Referent	Referent
II.2	-0.3	-0.5	-0.1	0.003
III.1	0.0	-0.4	0.5	0.9
III.2	0.0	-1.3	1.4	>0.09
**Number of Starts 3yo**				
I and II.1	Referent	Referent	Referent	Referent
II.2	-0.4	-0.7	-0.1	0.003
III.1	-0.7	-1.2	-0.2	0.004
III.2	-0.9	-1.9	0.1	0.09
**Number of Starts ≥ 4yo**				
I and II.1	Referent	Referent	Referent	Referent
II.2	-1.1	-2.1	-0.2	0.01
III.1	-2.9	-4.4	-1.4	<0.001
III.2	-4.6	-7.2	-2.0	0.001
**Number of Wins Career**				
I and II.1	Referent	Referent	Referent	Referent
II.2	-0.2	-0.4	-0.01	0.04
III.1	-0.4	-0.8	-0.09	0.01
III.2	-0.7	-1.4	0.01	0.05
**Number of Wins 2yo**				
I and II.1	Referent	Referent	Referent	Referent
II.2	0.0	-0.2	0.2	>0.9
III.1	-0.2	-0.5	0.1	0.1
III.2	0.6	-1.8	3.1	0.6
**Number of Wins 3yo**				
I and II.1	Referent	Referent	Referent	Referent
II.2	-0.1	-0.2	0.1	0.3
III.1	-0.1	-0.3	0.1	0.6
III.2	0.1	-0.5	0.7	0.7
**Number of Wins ≥ 4yo**				
I and II.1	Referent	Referent	Referent	Referent
II.2	-0.0	-0.2	0.2	0.7
III.1	-0.4	-0.7	0.0	0.05
III.2	-0.6	-1.3	0.1	0.1
**Number of Places Career**				
I and II.1	Referent	Referent	Referent	Referent
II.2	-0.5	-1.0	-0.1	0.02
III.1	-1.3	-2.1	-0.5	0.001
III.2	-2.3	-3.7	-1.0	<0.001
**Number of Places 2yo**				
I and II.1	Referent	Referent	Referent	Referent
II.2	-0.1	-0.3	0.1	0.4
III.1	-0.1	-0.5	0.2	0.4
III.2	0.6	-1.1	2.38	0.5

**Number of Places 3yo**				
I and II.1	Referent	Referent	Referent	Referent
II.2	-0.2	-0.3	0.0	0.06
III.1	-0.3	-0.6	0.0	0.05
III.2	-0.4	-1.1	0.2	0.2
**Number of Places ≥ 4yo**				
I and II.1	Referent	Referent	Referent	Referent
II.2	-0.3	-0.8	0.1	0.2
III.1	-1.1	-1.8	-0.3	0.004
III.2	-2.7	-3.6	-1.8	<0.001

† A coefficient >1 represents that many units increase in performance for that grade compared to the mean of grades I and II.1, whereas a coefficient <1 represents a decrease.
